# Suspected Cannabinoid Hyperemesis Syndrome in a Traveler from a Cannabis-Legal Country: A Case Report from Japan

**DOI:** 10.14789/ejmj.JMJ26-0010-CR

**Published:** 2026-06-30

**Authors:** SHIHO TSUGE, RIKI SAKURAI, KENTA KONDO, KATSUHIKO KADOTA, MAKOTO HIKI, SHIN WATANABE, YUTAKA KONDO

**Affiliations:** 1 Department of Emergency and Critical Care Medicine, Juntendo University Hospital, Tokyo, Japan; 1 Department of Emergency and Critical Care Medicine, Juntendo University Hospital, Tokyo, Japan

**Keywords:** cannabinoid hyperemesis syndrome (CHS), international traveler, cannabis regulation, case report

## Abstract

Cannabinoid hyperemesis syndrome (CHS) is characterized by recurrent nausea, vomiting, and abdominal pain in chronic cannabis users. We report a case of suspected CHS in a 37-year-old Canadian male who presented to a Japanese emergency department (ED) with severe abdominal pain and persistent vomiting. He reported chronic cannabis use two to four times per week for several years, with the last use the day before departure from Canada. Urine drug screening was positive for tetrahydrocannabinol. Although the clinical presentation was consistent with the hyperemetic phase of CHS, a definitive diagnosis could not be established due to the single-visit nature of the encounter and incomplete acquisition of key diagnostic information. This case highlights the diagnostic challenges of CHS in foreign travelers presenting to EDs in cannabis-prohibited countries, where linguistic, cultural, and legal barriers may impede timely recognition. Early consideration of CHS and systematic history-taking are essential to avoid diagnostic delays in this population.

## Introduction

Cannabinoid hyperemesis syndrome (CHS) is characterized by recurrent nausea, vomiting, and abdominal pain observed in patients with chronic and frequent cannabis use^1)^. With the global expansion of cannabis legalization, CHS-related emergency department (ED) visits have increased substantially, with an approximately five-fold rise reported in the United States between 2016 and 2022^2)^.

In Japan, where cannabis use remains strictly prohibited by law^3)^, clinical experience with CHS is limited, and to our knowledge, no domestic case reports have been published. Given the rapid increase in inbound travelers from cannabis-legal jurisdictions, emergency physicians in Japan may increasingly encounter CHS; however, the diagnostic challenges posed by linguistic, cultural, and legal barriers in such settings have not been adequately addressed.

We report this case to highlight the clinical and communicative difficulties associated with recognizing CHS in foreign travelers presenting to the ED in cannabis-prohibited countries.

## Case report

### Patient history

A 37-year-old Canadian male presented to the ED with severe abdominal pain and persistent vomiting. He had traveled from Canada to Japan for tourism two days prior and developed symptoms during the flight. As symptoms persisted intermittently without improvement, emergency medical services were contacted the day after arrival. His medical history included attention-deficit/hyperactivity disorder and depression, managed with selective serotonin reuptake inhibitors. He had no other significant medical history, with no history of sexually transmitted infections. There was no history of consuming raw or spoiled food, and no known exposure to infectious illness in his surroundings.

### Objective findings and diagnostic assessment

At the ED, he had a Glasgow Coma Scale score of 15 (E4V5M6); however, he was markedly agitated, standing up on the examination bed and verbally assaulting medical staff. Other vital signs were stable: heart rate 76 beats/min, respiratory rate 16 breaths/min, blood pressure 105/87 mmHg, temperature 36.6℃, and oxygen saturation 99% on room air. No abnormalities were identified on examination of the head and neck, chest, extremities, or neurological assessment. No injection marks were observed. Abdominal examination revealed diffuse tenderness without guarding, rebound tenderness, or abnormal bowel sounds. Laboratory findings are shown in Table 1. Venous blood gas analysis revealed respiratory alkalosis, suggesting hyperventilation due to agitation. Blood tests revealed neutrophilic leukocytosis, mildly elevated liver enzymes, and mildly elevated hemoglobin. Infectious etiology was not actively pursued given the absence of fever and negative C-reactive protein. Other parameters were within normal limits, and the observed abnormalities were attributed to dehydration and persistent vomiting. Abdominal ultrasonography revealed no findings suggestive of organic disease. A computed tomography (CT) scan was not performed as the patient refused due to concerns about insurance costs.

**Table 1:** Laboratory data on admission

Parameter	Patient value	Reference range
pH	7.6	
pvO_2_ (mmHg)	28.8	
pvCO_2_ (mmHg)	19.6	
Bicarbonate (mmol/L)	19.2	
Lactate (mmol/L)	3.3	
Glucose (mg/dL)	147	
White cell count (/μL)	15,700	3,900-9,700
Neutrophils (%)	89.1	37.9-76.8
Lymphocytes (%)	7.8	16.5-49.5
Hemoglobin (g/dL)	17.1	13.4-17.1
Total bilirubin (mg/dL)	1.2	0.4-1.2
AST (U/L)	45	5-37
ALT (U/L)	73	6-43
ALP (U/L)	69	38-113
LDH (U/L)	224	124-222
Amylase (U/L)	56	43-124
CK (U/L)	237	57-240
BUN (mg/dL)	17	9-21
Creatinine (mg/dL)	0.66	0.6-1.0
Sodium (mmol/L)	142	135-145
Potassium (mmol/L)	3.7	3.5-5
C-reactive protein (mg/dL)	0.18	-0.29

AST: Aspartate aminotransferase, ALT: Alanine aminotransferase, ALP: Alkaline phosphatase, LDH: Lactate dehydrogenase, CK: Creatine kinase, BUN: Blood urea nitrogen Reference ranges for venous blood gas parameters are not shown, as standardized reference ranges for venous samples were unavailable.

As no clear organic cause was identified to explain his agitation and aggression, drug intoxication was suspected. Upon further history-taking regarding substance use, he reported a history of chronic and regular cannabis use for several years, using cannabis two to four times per week, with his last use the day before his departure. Urine drug screening (SIGNIFY^TM^ ER kit) was positive for cannabis (tetrahydrocannabinol [THC]). Following a thorough review of his history and clinical presentation, CHS was suspected as the leading differential diagnosis, based on his history of chronic cannabis use, a positive urine drug screening for cannabis, and otherwise unexplained recurrent vomiting and abdominal pain.

### Treatment and clinical course

In the ED, 1000mg of intravenous acetaminophen was administered initially, without success. Subsequently, 15mg of pentazocine and 25mg of hydroxyzine hydrochloride were administered to improve abdominal pain, vomiting, and agitation. As symptoms sufficiently improved, the patient was discharged from the ED on the same day. At discharge, he was advised to abstain from cannabis use and informed of the possibility of symptom recurrence. Although we do not know when he returned to Canada, no subsequent hospital visits have been documented to our knowledge.

## Discussion

We report a rare and instructive case of suspected CHS in a foreign patient, highlighting the unique diagnostic challenges associated with differing linguistic, cultural, and legal backgrounds. This report underscores the importance of considering CHS in EDs within cannabis-prohibited countries.

CHS is a relatively new clinical entity first reported in Australia in 2004^4)^. Subsequently, the legalization of cannabis in the United States, Canada, and several other countries has led to a surge in reported cases and growing global awareness^5)^. CHS predominantly affects individuals aged 18-35 years, with males comprising approximately 69% of cases^1, 2, 6)^. The diagnosis of CHS is based on the Rome Ⅳ criteria, which require all three of the following to be fulfilled: (i) stereotypical episodic vomiting, (ii) onset following prolonged cannabis use, and (iii) resolution of symptoms with sustained cannabis cessation. These criteria must have been present for the preceding three months, with symptom onset occurring at least six months prior to diagnosis^7)^. Compulsive hot bathing behavior is a recognized supportive feature with diagnostic value^8)^.

The clinical presentation of this case, featuring severe vomiting and abdominal pain in the setting of chronic cannabis use, is consistent with the hyperemetic phase of CHS. However, a definitive diagnosis could not be established. While chronic cannabis use exceeding one year was confirmed, prior similar episodes could not be verified, and symptom resolution following cessation could not be observed. Notably, in chronic cannabis users, THC metabolites may remain detectable in urine for several weeks following cessation^9)^; therefore, the positive urine drug screen in this case does not necessarily confirm recent use and must be interpreted with caution. These findings illustrate the inherent difficulty of applying the Rome Ⅳ criteria in a single ED encounter, and the diagnosis remains that of suspected CHS. Several diagnostic challenges were encountered. Compulsive bathing behavior was not assessed due to insufficient clinical awareness of CHS at the time of evaluation. Additionally, the patient did not initially disclose cannabis use, and the diagnosis was only considered after a positive urine drug screening for THC. These issues reflect the combined impact of linguistic and cultural barriers and limited clinical recognition of CHS.

For acute pharmacological management, dopamine antagonists such as haloperidol and droperidol are recommended as first-line agents, with topical capsaicin as adjunctive therapy^10-13)^. Conventional antiemetics such as ondansetron have demonstrated limited efficacy, and opioids should be avoided due to the risk of exacerbating symptoms and the potential for dependence^1, 10, 13)^. The definitive treatment is complete cannabis cessation, after which symptoms typically resolve within weeks to months. However, recurrence is common, as many patients resume use due to dependence or social factors, and therefore, psychosocial support is recommended to improve long-term outcomes^14)^.

Regarding treatment in this case, pentazocine and hydroxyzine were administered concurrently for severe pain and agitation. In light of current evidence, the use of pentazocine, an opioid, was suboptimal; haloperidol would have been a more appropriate first-line agent^10, 13)^. Whether symptom improvement was attributable to hydroxyzine, pentazocine, or spontaneous resolution cannot be determined, and no evidence currently supports the use of hydroxyzine in CHS.

In cannabis-prohibited countries, foreign patients may face significant psychological barriers to disclosing cannabis use, even when such use was legal in their home country, and language barriers further compound these difficulties. In such settings, early consideration of CHS as a differential diagnosis and systematic history-taking —including specific inquiry about bathing behavior— is essential for timely recognition. These challenges are not limited to individual clinicians but represent a broader structural issue for emergency healthcare systems in cannabis-prohibited countries.

There are several limitations to this report. The single ED visit, incomplete acquisition of key diagnostic information, inability to perform advanced imaging, and unknown long-term outcomes precluded a definitive diagnosis and limited the generalizability of our findings.

## Conclusion

While CHS remains rare in Japan owing to strict regulations, the increasing global mobility of travelers necessitates that physicians in non-legalized countries maintain a high index of suspicion for this condition. Overcoming language barriers and low clinical awareness is essential for the timely recognition of CHS. Even when long-term follow-up is impossible, providing a provisional diagnosis and education regarding cannabis cessation constitutes a vital medical intervention for international travelers.

## Author contributions

ST wrote the manuscript and reviewed the literature. RS, KK, KK, and MH performed the clinical treatment. YK and SW provided critical advice on the manuscript and supervised the writing process. All authors read and approved the final manuscript.

## Data availability

As this study is a single-patient case report, no datasets were generated or analyzed.

## Conflicts of interest statement

The authors declare that there are no conflicts of interest.

## Informed consent

Oral informed consent was obtained from the patient for the publication of this case report.
